# Scaling and Volatility of Breakouts and Breakdowns in Stock Price Dynamics

**DOI:** 10.1371/journal.pone.0082771

**Published:** 2013-12-23

**Authors:** Lu Liu, Jianrong Wei, Jiping Huang

**Affiliations:** Departement of Physics and State Key Laboratory of Surface Physics, Fudan University, Shanghai, China; Queen’s University Belfast, United Kingdom

## Abstract

**Background:**

Because the movement of stock prices is not only ubiquitous in financial markets but also crucial for investors, extensive studies have been done to understand the law behind it. In particular, since the financial crisis in 2008, researchers have a more interest in investigating large market volatilities in order to grasp changing market trends.

**Methodology/Principal Findings:**

In this work, we analyze the breakouts and breakdowns of both the Standard & Poor’s 500 Index in the US stock market and the Shanghai Composite Index in the Chinese stock market. The breakout usually represents an ongoing upward trend in technical analysis while the breakdown represents an ongoing downward trend. Based on the renormalization method, we introduce two parameters to quantize breakouts and breakdowns, respectively. We discover scaling behavior, characterized by power-law distributions for both the breakouts and breakdowns in the two financial markets with different power-law exponents, which reflect different market volatilities. In detail, the market volatility for breakdowns is usually larger than that for breakouts. Moreover, as an emerging market, the Chinese stock market has larger market volatilities for both the breakouts and breakdowns than the US stock market (a mature market). Further, the short-term volatilities show similar features for both the US stock market and the Chinese stock market. However, the medium-term volatilities in the US stock market are almost symmetrical for the breakouts and breakdowns, whereas those in the Chinese stock market appear to be asymmetrical for the breakouts and breakdowns.

**Conclusions/Signicance:**

The methodology presented here provides a way to understand scaling and hence volatilities of breakouts and breakdowns in stock price dynamics. Our findings not only reveal the features of market volatilities but also make a comparison between mature and emerging financial markets.

## Introduction

Because the movement of stock prices is a key topic in financial markets, it has attracted extensive attention from not only market traders but also researchers. Since the outset of the stock market, economists and market traders have summarized numerous qualitative theories of stock price movements [1], [2], [3], [4]. The theory of technical analysis [5], [6], [7] containing typical price movement patterns based on empirical financial market data is widely used today by those chart holders to predict future market trends [8], [9].

In this work, we mainly focus on the statistical properties of stock price movements and attempt to give quantificational analysis. In fact, a lot of statistical analyses have been applied to the study of financial markets. In 1995, Mantegna and Stanley analyzed the probability distribution for a particular economic index and found its scaling behaviour [10]. Apart from the scaling behaviour, based on the empirical data of prices researchers have also discovered many properties of stock prices named as stylized facts [11], [12], [13], such as volatility clustering [14], [15], fat tails in the distribution of returns [16],[17],[18] and long-term correlations of stock prices [19], [20], [21]. Clearly, these discoveries are still far from enough for people to fully understand the mechanism behind stock markets. In fact, since the worldwide financial crisis in 2008, the society have paid more and more attentions to the study of large market volatilities. For example, Johnson and Sornette (2010) [22] studied the financial bubble caused by speculators and worldwide crises as a result of herding [23]. Preis et al. (2011) investigated switching processes in financial markets and explained catastrophic bubbles in the 2008 financial crisis [24]. Accordingly, here we extend the statistical methods to treat large fluctuations of stock prices, particularly the breakouts and breakdowns in the stock price movements, which is crucial and practical for technical analyzers to understand market trends [25], [26]. We will apply the renormalization method [24], [27], [28] and introduce two parameters, *R* and *R′*, to respectively quantify the breakouts and breakdowns, in order to illustrate volatilities in stock price movements. For this purpose, we have collected the data of stock prices from both the Standard & Poor’s 500 Index (S&P500 Index) in the US stock market (a mature market) and the Shanghai Composite Index (SH Index) in the Chinese stock market (an emerging market). This also allows us to compare similarities and differences between the two markets.

## Methods

A new price higher than the previous maximum always indicates an upward trend, while a new price lower than the previous minimum indicates an downward trend. These two phenomena are usually defined as breakouts and breakdowns in the financial market.

**Figure 1:**
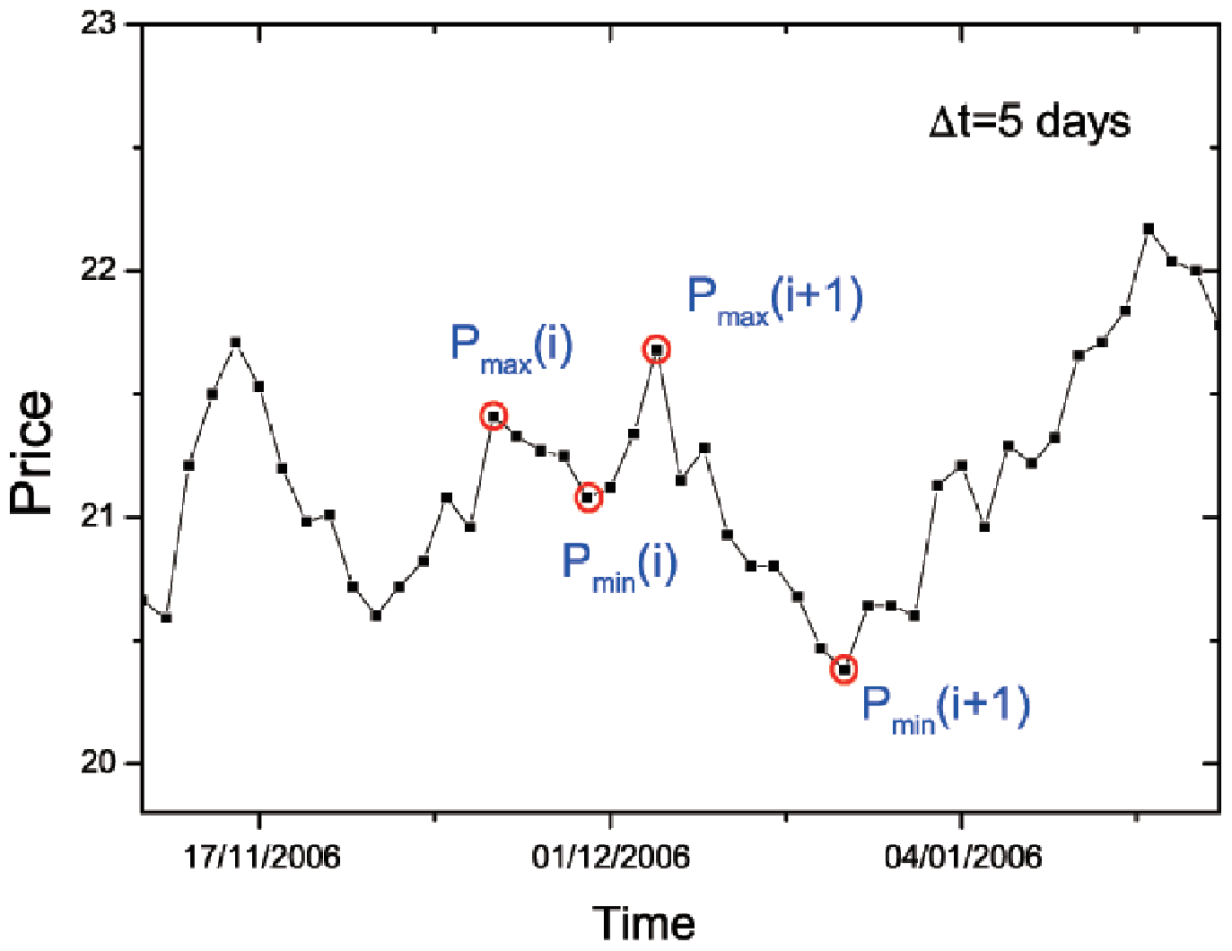
Schematic graph showing the breakout and breakdown. Square symbols denote the daily closing price versus time, for one component stock of S&P500 Index ranged from November 11, 2006 to January 22, 2007. *P*_max_(*i*) and *P*_max_(*i*+1) are the local maxima in the time interval [*t*–Δ*t*, *t*+Δ*t*] and *P*_min_(*i*) is the local minimum between the two adjacent local maxima. Here Δ*t* = 5 days. The line is a guide to the eye.

Here we introduce two dimensionless parameters, *R* and *R*′, by means of the renormalization method to quantize the breakouts and breakdowns, respectively. Because the movement of stock prices yields a time series of prices, we denote the stock price at time *t* as *P*(*t*), where *t* = 1, 2, 3, …, *N*. When given a time duration Δ*t*, we denote the highest stock price in the time interval [*t*–Δ*t*, *t*+Δ*t*] as the local maximum *P*_max_. Moving a window with the length of 2Δ*t*, we can get a series of local maxima *P*_max_(*i*), where *i* = 1, 2, 3, …, *N* [28]. After that, we define the local minimum *P*_min_(*i*) as the lowest stock price between two adjacent local minima *P*_max_(*i*) and *P*_max_(*i*+1). Thus we also get a series of local minima *P*_min_(*i*), *i* = 1, 2, 3, …, *N*–1. See Fig. 1 that contains an example for Δ*t* = 5 days.

Then, the renormalized breakout, *R*(*i*), is defined as
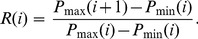
(1)


Clearly *R* represents the intensity of each breakout. According to the definition of breakouts, *R* is always larger than one.

Similarly, we define the renormalized breakdown, *R*′(*i*), as
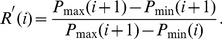
(2)


Here *R*′ stands for the intensity of each breakdown, and the definition of breakdowns also means that *R*′ should be always larger than one as well. Apparently, both *R* and *R*′ represent large price movements, and they are certainly indicative of ongoing trends of stock prices.

## Results and Discussion

### (1) Statistical Analysis of Breakouts (*R*) and Breakdowns (*R*′)

To study the breakouts and breakdowns of the US stock market, we calculate all the 500 stocks used for calculating the S&P500 Index. The data of daily closing prices for each stock date back from its starting point of issue till July 31, 2012. To study the breakouts and breakdowns in the Chinese stock market as a comparison, we collect daily closing prices of 847 stocks used for calculating the SH Index and the data for each stock date back from its starting point of issue till July 31, 2012. For the Chinese stock market, here we only include stocks with more than three months of data. First, let us take a glimpse at the data of *R* and *R*′ from both the 500 stocks for the S&P500 and the 847 stocks for the SH Index. We calculate the mean value and standard deviation of *R* and *R*′; see Table 1. As we can see, for both the S&P500 and SH Index, the standard deviation of *R* is smaller than that of *R*′. That is, in average, the market experiences higher volatility when the price is moving down than when the price is moving up. Then let us look at the two financial markets separately. The mean values and standard deviations of *R* and *R*′ in the Chinese stock market are both larger than those in the US stock market, indicating that the emerging market (Chinese stock market) is less stable and always experiences larger price movements.

**Table 1:** Mean value and standard deviation of breakouts (*R*) and breakdowns (*R*′).

		Mean	Std. Dev.
**500 stocks of the S&P500 Index**	*R*	3.16064	6.19859
	*R*′	2.77648	6.9888
**847 stocks of the SH Index**	*R*	3.8049	9.3666
	*R*′	3.09	9.71

After a brief statistical analysis of *R* and *R*′, we are now in a position to investigate the cumulative distribution functions (CDFs).

### (2) CDF of Breakouts (*R*) and Breakdowns (*R*′)

The CDF, *F_X_*(*x*), for a discrete variable *X* describes the probability distribution of *X* to be found larger than or equal to a number *x* [29], [30]. It is also named as the complementary cumulative distribution function or tail distribution. *F_X_*(*x*) is defined for every number *x* as
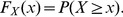
(3)


Every CDF is monotonically decreasing. If we define *F_X_*(*x*) for any positive real number *x*, then *F_X_*(*x*) has two properties:
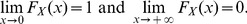
(4)


To comply with our notations, here *X* represents *R* or *R*′. Because both *R* and *R*′ are always larger than one, their CDFs only have positive tails.

**Figure 2:**
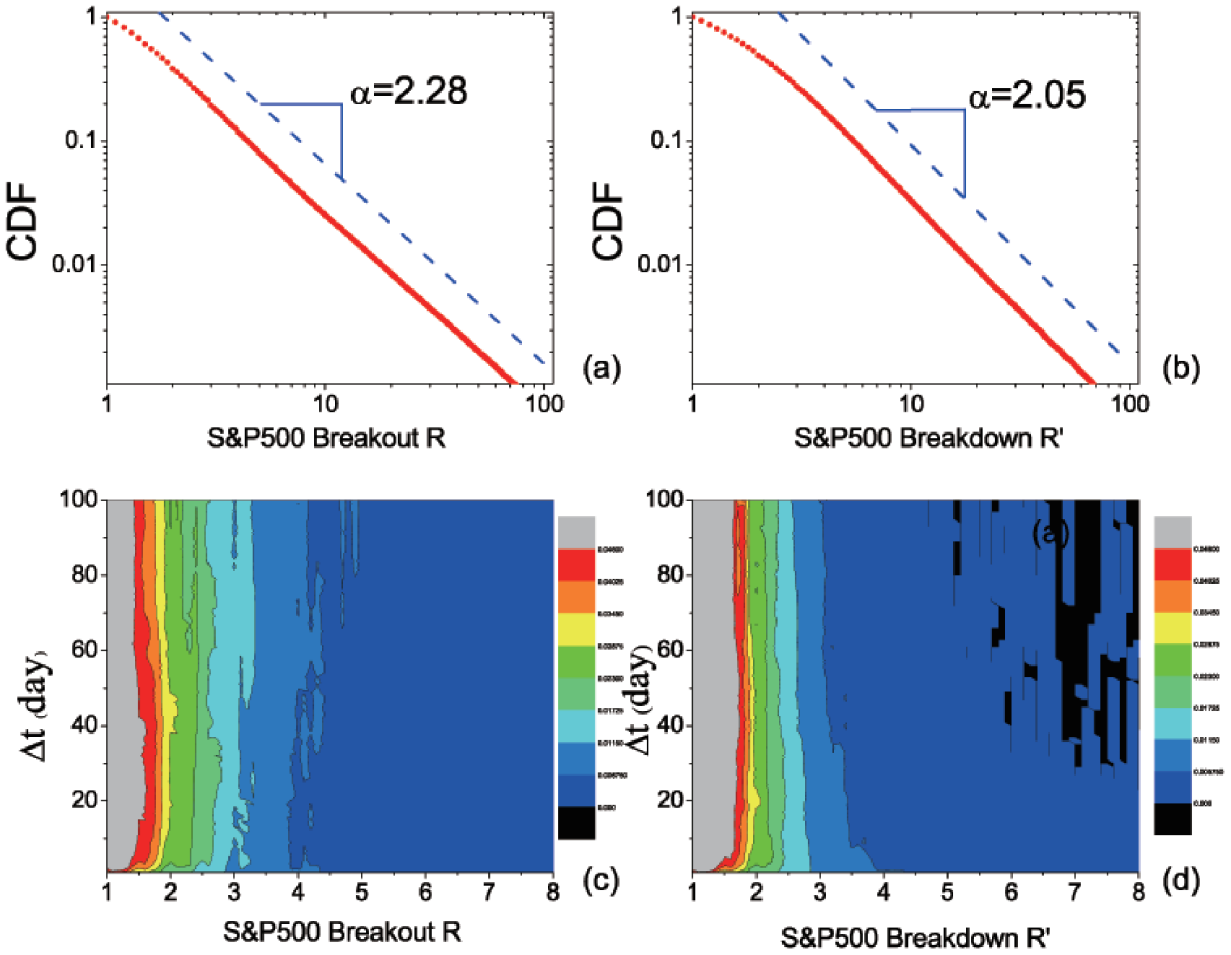
Distribution functions of *R* and *R*′ for the S&P500 Index in the US stock market: CDF of (a) *R* and (b) *R*′; PDF of (c) *R* and (d) *R*′ for different time intervals, Δ*T*. The curve in either (a) or (b) appears to be a straight line in the log-log plot, which is indicative of a power-law fitting. The dash line is a guide to the eye. Regarding the fitting, more details are listed in Table 2.

**Figure 3:**
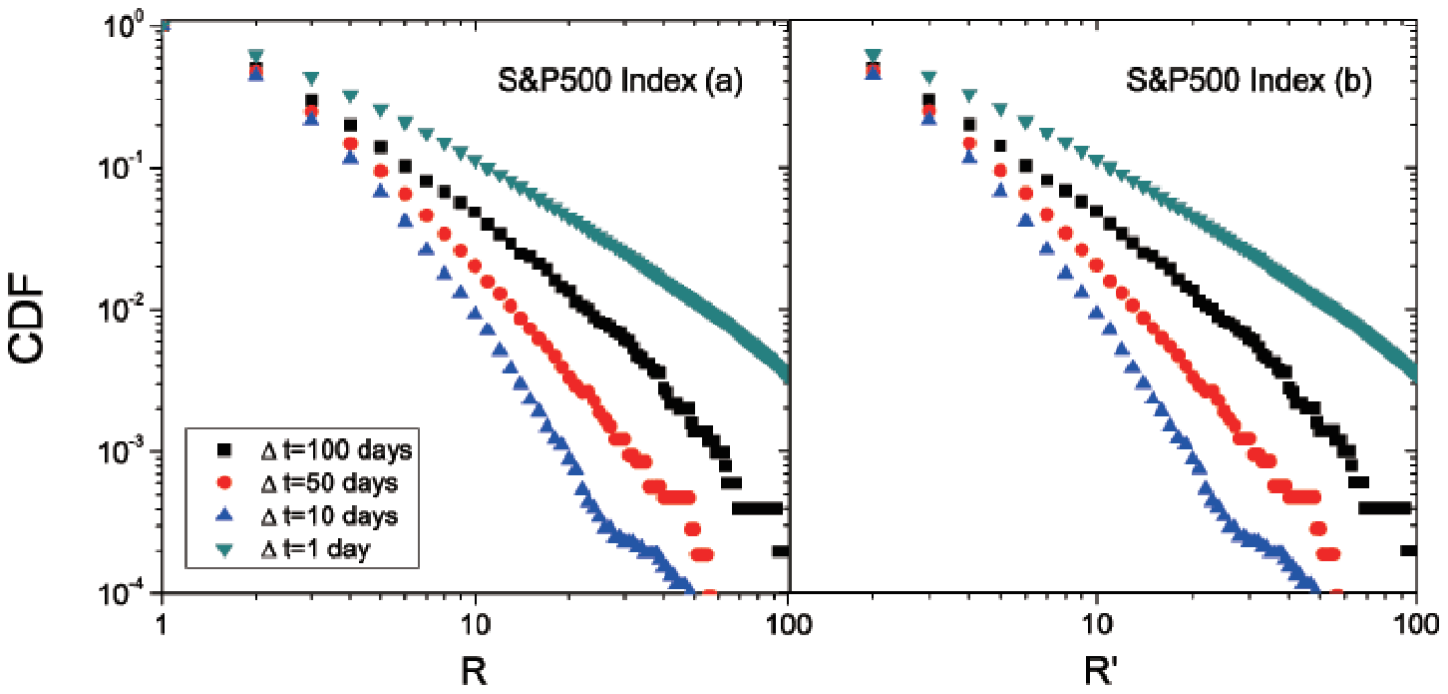
CDFs of (a) *R* and (b) *R*′ for different time intervals, Δ*T*, for the S&P500 Index in the US stock market. Each curve can be fitted with a power law, as indicated by the straight line in the log-log plot. Regarding the fitting, more details are listed in Table 2.

First we analyze the CDF of S&P500. The overall time interval Δ*t* is from 1 day to 100 days. As is shown in Fig. 2(a–b), the CDFs of breakouts and breakdowns are almost straight lines in the log-log plot, indicating a power-law distribution like *P*(*x*)∼*x* ^–*α*^. The exponent *α* is 2.28 for breakouts and 2.05 for breakdowns. More information on the fitting can be found in Table 2. The smaller *α* of breakdowns shows that the downward trend of stock prices usually experiences larger price movements than the upward trend. This echoes with the previous analysis about Table 1. To further study the distribution of *R* and *R*′ for each Δ*t*, we compute the probability distribution function (PDF) of each Δ*t* separately; see Fig. 2(c–d). Clearly, for each Δ*t*, the PDF generally decays as the breakout *R* or breakdown *R*′ increases. Thus the distributions of breakouts and breakdowns of different Δ*t* show similar tendency. Particularly, we find almost the same power-law distribution of CDFs for *R* and *R*′ at each Δ*t*; see Fig. 3. In Fig. 3(a–b), we pick up the CDFs of four typical time durations. As the time duration increases, they all show a straight line in the log-log plot, while the exponent *α* is quite different for different time intervals. The corresponding parameters related to the power-law fitting are included in Table 2. Interestingly, the renormalized breakout, *R*, of Δ*t* = 1 day has the slowest attenuation as it has the smallest exponent, 1.41, whereas *R* for the intervals of larger Δ*t* (10, 50, or 100 days) decays more rapidly. This indicates that short-term (Δ*T = *1 day) investments have higher market volatility than the mid-term (Δ*T = *10, 50, and 100 days) investments. Similar results hold for the renormalized breakdown, *R*′. Particularly, the CDFs of different time intervals (Δ*T*) are almost symmetrical for the breakouts (*R*) and breakdowns (*R*′) in the US stock market. That is, there is almost the same market volatility in the downward and upward trends.

**Table 2:** The parameters for fitting CDFs shown in Figs. 2 and 3 (S&P500 Index in the US stock market) with *R* or *R*′ ranging from “Minimum” to “Maximum”.

CDF	Minimum	Maximum	*α*	Std. Dev.	*r* ^2^
**Fig. 2(a)** **:** *R*	1	100	2.28	0.01	0.99
**Fig. 2(b)** **:** *R*′	1	100	2.05	0.01	0.99
Fig. 3(a): *R*for Δ**t = 1**	2	62	1.41	0.01	0.99
Fig. 3(a): *R*for Δ**t = 10**	2	28	3.28	0.02	0.99
Fig. 3(a): *R*for Δ**t = 50**	2	50	2.52	0.01	0.99
Fig. 3(a): *R*for Δ**t = 100**	2	62	1.86	0.01	0.99
Fig. 3(b): *R*′for Δ**t = 1**	3	58	1.39	0.01	0.99
Fig. 3(b): *R*′for Δ**t = 10**	3	27	3.15	0.03	0.99
Fig. 3(b): *R*′for Δ**t = 50**	3	53	2.52	0.01	0.99
Fig. 3(b): *R*′for Δ**t = 100**	3	58	1.87	0.01	0.99

*α* is the exponent (scaling parameter); “Std. Dev.” is the standard deviation for *α*, indicating the fitting error; and *r* ^2^ is the regression coefficient that represents the degree of fitting with the power law: the perfect fitting corresponds to *r* ^2^ = 1 [30].

**Figure 4:**
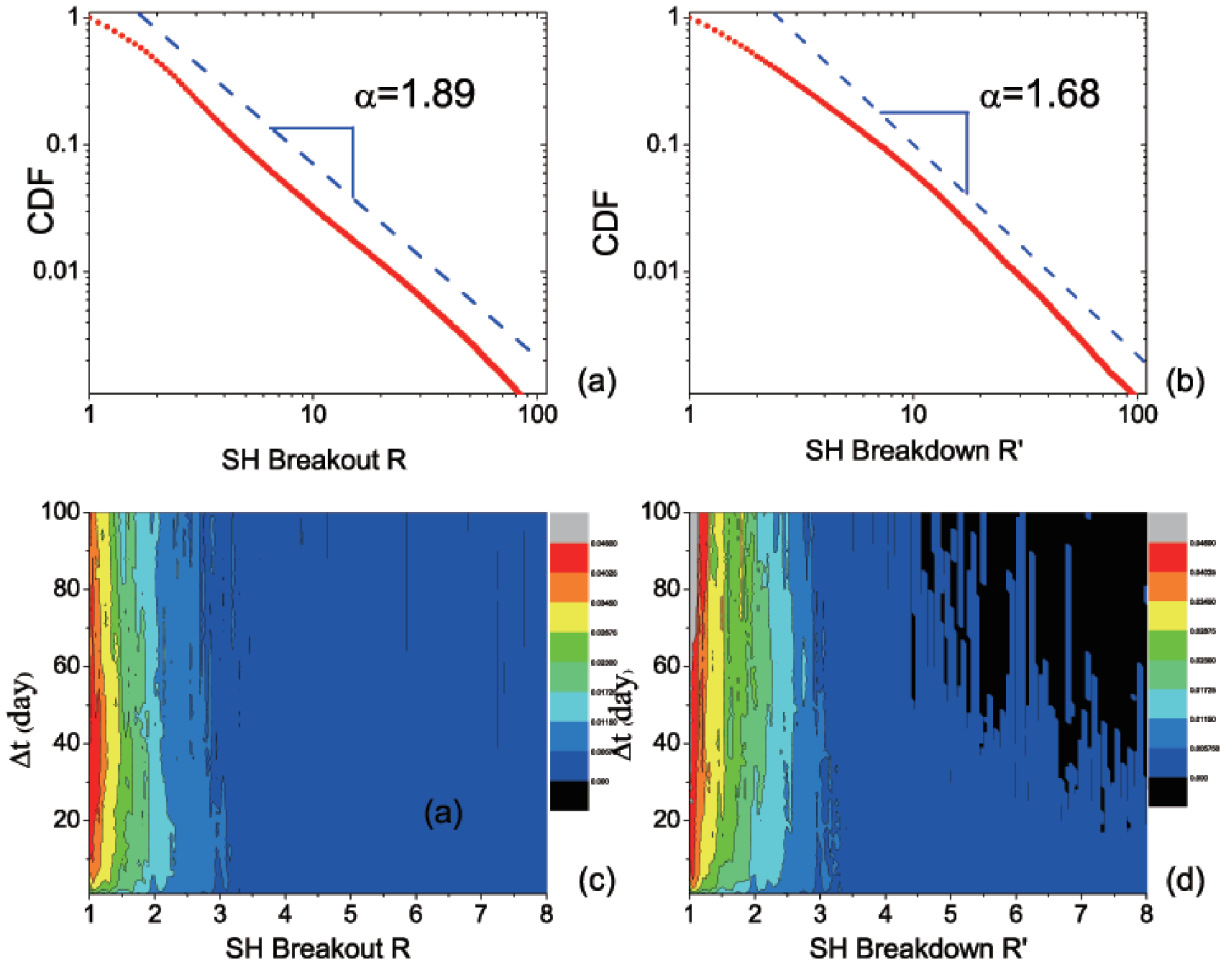
Same as Fig. 2, but for the SH Index in the Chinese stock market. The dash line is a guide to the eye. Regarding the fitting, more details are listed in Table 3.

**Figure 5:**
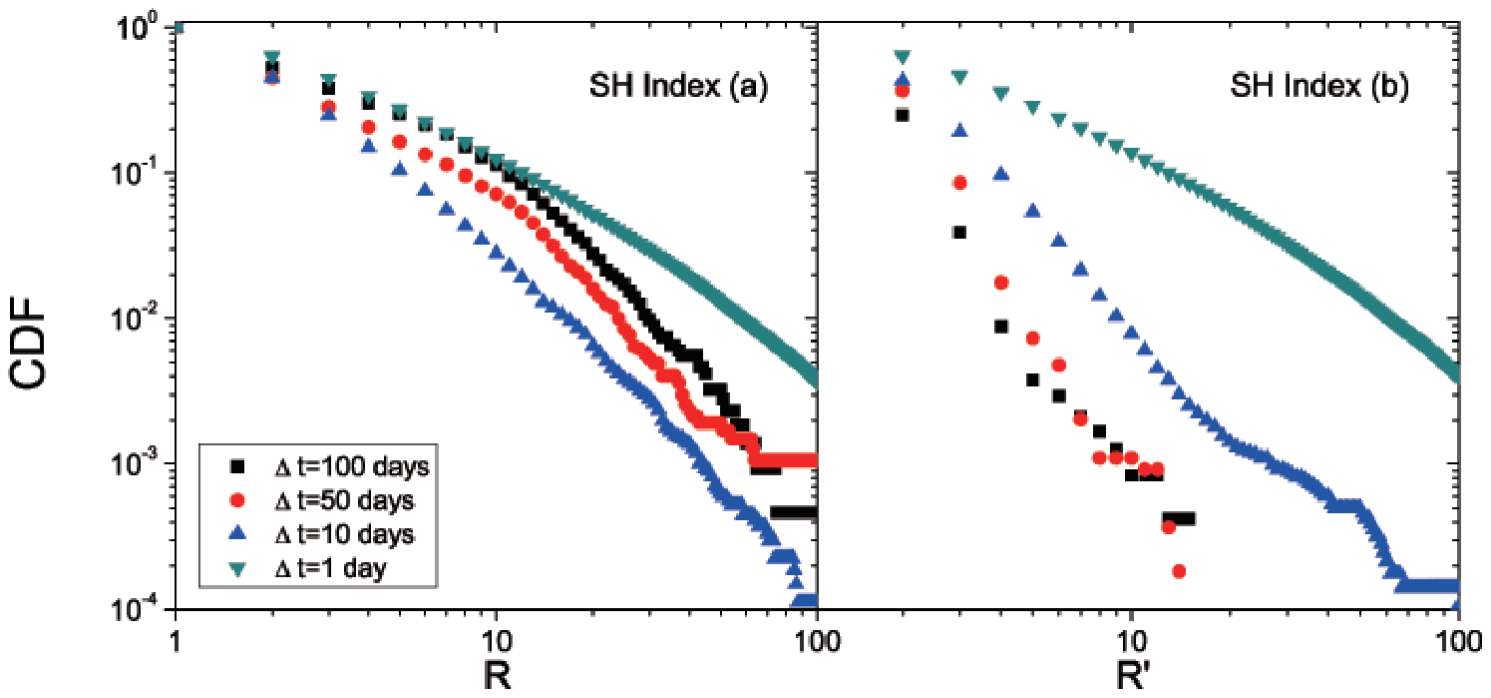
Same as Fig. 3, but for the SH Index in the Chinese stock market. Regarding the fitting, more details are listed in Table 3.

After investigating the price distribution of the US stock market, we further compare the results in the Chinese stock market. We apply the same calculations to study the CDFs and PDFs of breakouts *R* and breakdowns *R*′ for the 847 stocks of the SH Index. The overall CDF of renormalized breakdowns and breakouts for the SH Index both show a straight line in the log-log plot, indicating a power-law distribution *P*(*x*)∼*x* ^–*α*^ as well; see Fig. 4(a–b). The exponent *α* is 1.89 for breakouts and 1.68 for breakdowns. Corresponding parameters for fitting are listed in Table 3. Similar to the 500 stocks of the S&P500 Index, breakdown *R*′ has a smaller *α* ( = 1.68). Particularly, the *α*’s of both breakouts and breakdowns are smaller than those of the S&P500 Index, suggesting that as an emerging stock market, the SH Index has greater volatilities of price movements. Then for each Δ*t*, the PDFs of breakdowns and breakouts both decay as *R* and *R*′ increase; see Fig. 4(c–d). Further, as shown in Fig. 5(a–b), similar to the result of the S&P500 Index, we observe that the CDFs of breakouts and breakdowns in the short term (Δ*T* = 1 day) decays slower than in the medium term (Δ*T* = 10, 50, and 100 days). However, unlike the result of the S&P500 Index, the CDFs of breakouts and breakdowns in the SH Index with large intervals (Δ*T* = 10, 50, and 100 days) show different price movements, which appear to be asymmetrical. This reflects a difference between the US stock market and the Chinese stock market. An explanation might be that there are many new issues in the Chinese stock market and then the medium-term data are insufficient [31]. Further as an emerging market, stocks in the Chinese market may experience more external interferences such as government regulations. Thus the asymmetrical distribution of breakouts and breakdowns in the SH Index may be a reflection of this immature and growing financial market.

**Table 3:** The parameters for fitting CDFs shown in Figs. 4 and 5 (SH index in the Chinese stock market) with *R* or *R*′ ranging from “Minimum” to “Maximum”.

CDF	Minimum	Maximum	*α*	Std. Dev.	*r* ^2^
**Fig. 4(a)** **:** *R*	1	100	1.89	0.01	0.99
**Fig. 4(b)** **:** *R*′	1	100	1.68	0.01	0.99
Fig. 5(a): *R*for Δ**t = 1**	11	63	1.41	0.01	0.99
Fig. 5(a): *R*for Δ**t = 10**	11	63	2.59	0.03	0.99
Fig. 5(a): *R*for Δ**t = 50**	11	63	2.36	0.03	0.99
Fig. 5(a): *R*for Δ**t = 100**	11	63	2.22	0.03	0.99
Fig. 5(b): *R*′for Δ**t = 1**	2	40	1.19	0.01	0.99
Fig. 5(b): *R*′for Δ**t = 10**	2	40	2.95	0.02	0.99
Fig. 5(b): *R*′for Δ**t = 50**	2	14	5.27	0.11	0.99
Fig. 5(b): *R*′for Δ**t = 100**	2	15	6.44	0.09	0.99

*α* is the exponent (scaling parameter); “Std. Dev.” is the standard deviation for *α*, indicating the fitting error; and *r* ^2^ is the regression coefficient that represents the degree of fitting with the power law: the perfect fitting corresponds to *r* ^2^ = 1 [30].

## Conclusions

In conclusion, based on the renormalization method, we have introduced two parameters, *R* and *R*′, to quantize the breakouts and breakdowns of stock prices, respectively. We have analyzed both 500 stocks of the S&P500 Index in the US stock market and 847 stocks of the SH Index in the Chinese stock market, and discovered scaling behavior, characterized by power-law distributions for both *R* and *R*′ in the two financial markets with different power-law exponents, which represent different market volatilities. Particularly, the market volatility is commonly larger when the market is facing a downward pressure than an upward pressure (as *R*′ is larger than *R*). Moreover, as an emerging market, the Chinese stock market has larger market volatilities for the breakouts and breakdowns than the US stock market as a mature market because both *R* and *R*′ of the Chinese market are larger. Further, the market volatilities show similar features in the short term (Δ*t* = 1 day) for both the US stock market and the Chinese stock market. However, for the medium term (Δ*t* = 10, 50, or 100 days), volatilities in the US stock market are almost symmetrical for the breakouts and breakdowns, while the volatilities for the breakouts and breakdowns in the Chinese stock market appear to be asymmetrical. This indicates their distinct market characteristics. Our findings could provide a comprehensive understanding of volatilities related to breakouts and breakdowns of stock prices.
